# Pleiotrophin Deficiency Induces Browning of Periovarian Adipose Tissue and Protects against High-Fat Diet-Induced Hepatic Steatosis

**DOI:** 10.3390/ijms22179261

**Published:** 2021-08-26

**Authors:** Agata Zuccaro, Begoña Zapatería, María Gracia Sánchez-Alonso, María Haro, María Limones, Gloria Terrados, Adriana Izquierdo, Patricia Corrales, Gema Medina-Gómez, Gonzalo Herradón, Julio Sevillano, María del Pilar Ramos-Álvarez

**Affiliations:** 1 Department of Chemistry and Biochemistry, Facultad de Farmacia, Universidad San Pablo-CEU, CEU Universities, 28925 Alcorcón, Spain; agata.zuccaro@ceu.es (A.Z.); beg.zapateria.ce@ceindo.ceu.es (B.Z.); msancheza@ceu.es (M.G.S.-A.); maria.harogarcia@ceu.es (M.H.); maria.limonescornejo@ceu.es (M.L.); gloriamaria.terradosaguado@ceu.es (G.T.); pramos@ceu.es (M.d.P.R.-Á.); 2 Department of Basic Sciences of Health, Facultad Ciencias de la Salud, Universidad Rey Juan Carlos, 28922 Alcorcón, Spain; adriana.izquierdo@urjc.es (A.I.); patricia.corrales@urjc.es (P.C.); gema.medina@urjc.es (G.M.-G.); 3 Department of Pharmaceutical and Health Sciences, Facultad de Farmacia, Universidad San Pablo-CEU, CEU Universities, 28925 Alcorcón, Spain; herradon@ceu.es

**Keywords:** pleiotrophin, high-fat diet, periovarian AT, steatosis, liver metabolism, browning

## Abstract

(1) Background: Pleiotrophin preserves insulin sensitivity, regulates adipose tissue lipid turnover and plasticity, energy metabolism and thermogenesis. The aim of this study was to determine the role of pleiotrophin in hepatic lipid metabolism and in the metabolic crosstalk between the liver and brown and white adipose tissue (AT) in a high-fat diet-induced (HFD) obesity mice model. (2) Methods: We analyzed circulating variables, lipid metabolism (hepatic lipid content and mRNA expression), brown AT thermogenesis (UCP-1 expression) and periovarian AT browning (brown adipocyte markers mRNA and immunodetection) in *Ptn*^−/−^ mice either fed with standard-chow diet or with HFD and in their corresponding *Ptn*^+/+^ counterparts. (3) Results: HFD-*Ptn^−/−^* mice are protected against the development of HFD-induced insulin resistance, had lower liver lipid content and lower expression of the key enzymes involved in triacylglycerides and fatty acid synthesis in liver. HFD-*Ptn*^−/−^ mice showed higher UCP-1 expression in brown AT. Moreover, *Ptn* deletion increased the expression of specific markers of brown/beige adipocytes and was associated with the immunodetection of UCP-1 enriched multilocular adipocytes in periovarian AT. (4) Conclusions: *Ptn* deletion protects against the development of HFD-induced insulin resistance and liver steatosis, by increasing UCP-1 expression in brown AT and promoting periovarian AT browning.

## 1. Introduction

Obesity is a chronic disease associated with the regulation of both lipidic and glycemic metabolism and is defined by a body mass index (BMI) > 30 kg/m^2^ [1]. In addition, obesity is associated with the development of metabolic syndrome, type 2 diabetes mellitus, non-alcoholic fatty liver disease (NAFLD), hypertension, hyperlipidemia, and cardiovascular disease [2].

A high-fat diet, usually observed in obesity, increases the risk of developing primary hepatic steatosis, as overnutrition and lack of exercise cause the liver and other tissues to store the excess of energy as a short-term protective mechanism [2,3]. However, this protective mechanism in the liver is long-term associated with the development of NAFLD [4].

Recent studies have indicated that the increased metabolic activity of brown adipose tissue (BAT) may represent a novel therapeutical approach to prevent the development of NAFLD [5,6] and to reduce circulating lipids [7] due to the metabolic crosstalk between liver and BAT [8]. Moreover, in the last decade, several studies have highlighted the emergence of brown adipocytes in the white adipose depots in response to exercise [9], cold exposure [10], dietary factors [11] and cytokines or pharmaceuticals [12,13]; these brown cells within white adipose tissue can also contribute to increase energy expenditure and improve metabolic health.

Pleiotrophin (PTN) is a heparin-binding growth factor discovered for the first time to play a fundamental role in the early stages of development. PTN expression is strictly restricted in adulthood, apart from those processes that involve tissue regeneration, cell growth and angiogenesis [14,15,16,17]. Moreover, the gene encoding pleiotrophin (*Ptn*) is a proto-oncogene, and subsequently the levels of this protein are increased in tumour and neoplastic processes [15]. Furthermore, PTN has an inhibitory role in the in vitro differentiation of pre-adipocytes, suggesting a role for PTN as a regulator of adipogenesis [18,19], a process required to sequester lipids avoiding liver lipotoxicity [20]. In a previous study from our group, we reported that *Ptn* deletion modulates adiposity and fat distribution in rodents and is associated with an increased cold-induced thermogenesis contribution to energy expenditure. Furthermore, body temperature and the activity and expression of deiodinase, T3 and mitochondrial thermogenic activity in the brown adipose tissue of *Ptn*^−/−^ mice were higher than in wild-type controls [21]. BAT differentiation has also been shown to be regulated by changes in *Ptn* expression. In fact, in vitro treatment of mouse brown adipocytes (mBAs) with recombinant PTN diminished the expression of brown fat markers (*Cidea*, *Prdm16* and *Pgc1-α*), suggesting an inhibitory role of this cytokine in brown fat differentiation and thermogenesis [21].

PTN presents a mitogenic role in the liver, both in the development and in the regeneration of the hepatic tissue [22,23]. Although, in a recent study of our group, we have observed that pleiotrophin is an important player in maintaining hepatic metabolic homeostasis during late gestation [24], the role of PTN in hepatic metabolism in non-pregnant animals is unknown.

To fill this gap, the aim of this study was, using a *Ptn* knock-out mice model, to determine the role of pleiotrophin in hepatic lipid metabolism and the metabolic crosstalk between the liver and BAT in high-fat diet-induced (HFD) obesity. In the present study, we provide evidence that *Ptn* deletion protects against the development of HFD-induced insulin resistance and liver steatosis, by increasing UCP-1 expression in BAT and inducing periovarian adipose tissue browning.

## 2. Results

### 2.1. Effects of Ptn Deletion and High-Fat Diet Feeding on Body Weight and White and Brown Adipose Tissue Weights

**Figure 1:**
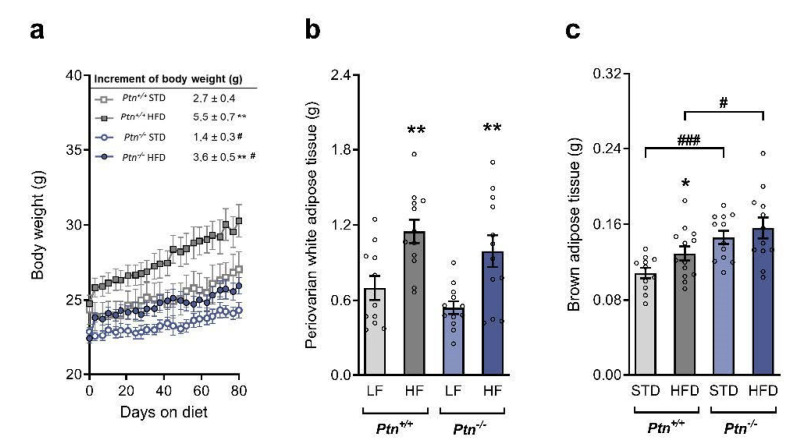
Effects of *Ptn* deletion and HFD feeding on body weight and periovarian and brown adipose tissue weights. (**a**) Body weight and increase in body weight (insert), (**b**) periovarian white adipose tissue weight and (**c**) brown adipose tissue weight of *Ptn^+/+^* and *Ptn^−/−^* mice fed with a standard chow diet (STD) or with a high-fat diet (HFD). Data are expressed as mean ± SEM of n = 12 mice/group. * *p* < 0.05; ** *p* < 0.01 for differences in the effect of HFD feeding within *Ptn^+/+^* or *Ptn^−/−^* mice. ^#^ *p* < 0.05; ^###^ *p* < 0.001 for differences between *Ptn^+/+^* and *Ptn^−/−^*.

Before the administration of the diet, *Ptn^−/−^* mice exhibited lower body weight than *Ptn^+/+^* mice. After 80 days on a high-fat diet (HFD), the body weight of HFD-*Ptn^−/−^* mice and HFD-*Ptn^+/+^* mice were markedly increased compared with the body weight of the animals fed with standard chow diet (STD) (Figure 1a). However, the increase in body weight during the experiment was smaller in the *Ptn^−/−^* than in *Ptn^+/+^* animals, regardless of the diet they received (Figure 1a, insert).

We next analyzed the periovarian adipose tissue weight. As shown in Figure 1b, the weight of this white adipose tissue (WAT) increases in both genotypes by the effect of the HFD, but no changes were observed between *Ptn^−/−^* and *Ptn^+/+^* mice (Figure 1b). Feeding with HFD significantly increased the weight of brown adipose tissue (BAT) in *Ptn^+/+^* mice (Figure 1c). However, although the weight of BAT did not increase in the HFD-*Ptn^−/−^* mice, BAT weight was significantly higher in the *Ptn^−/−^* mice than in the *Ptn^+/+^* mice, no matter which diet they were receiving. No differences were observed in the daily food intake either in the STD (3.27 ± 0.22 and 2,83 ± 0.12 for *Ptn^+/+^* and *Ptn^−/−^* mice, respectively, *p* = 0.20) or in the HFD-fed mice (2.57 ± 0.08 and 2.34 ± 0.16 for *Ptn^+/+^* and *Ptn^−/−^* mice, respectively, *p* = 0.16).

### 2.2. Ptn^−/−^ Mice Show Reduced Liver Weight and Altered Lipid Profile when Fed with a High-Fat Diet

**Figure 2:**
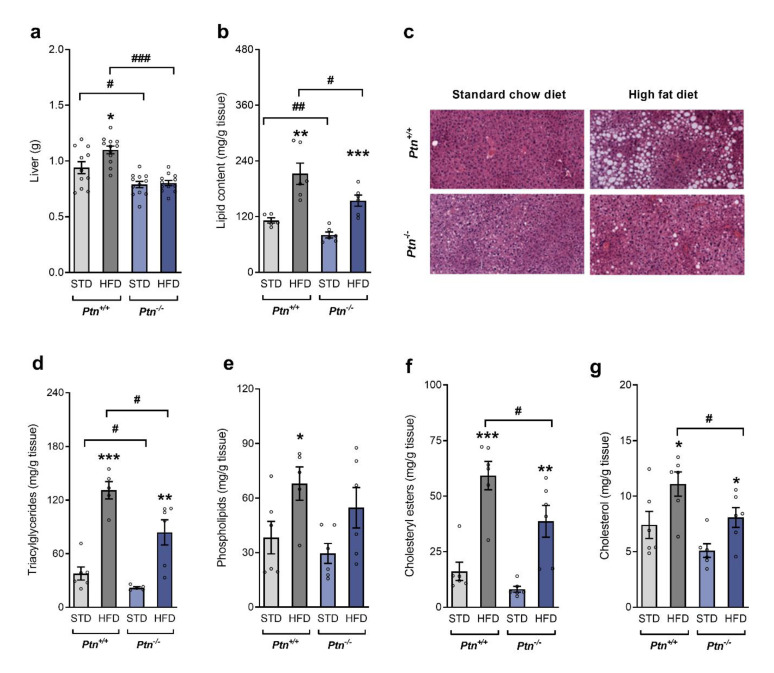
*Ptn*^−/−^ mice show reduced liver weight and altered lipid profile when fed with a high-fat diet. (**a**) Liver weight, (**b**) total hepatic lipid content, (**c**) hematoxylin-eosin staining of formalin-fixed paraffin embedded liver tissue sections, (**d**) hepatic triacylglycerides, (**e**) hepatic phospholipids, (**f**) hepatic cholesteryl esters, and (**g**) hepatic cholesterol of *Ptn^+/+^* and *Ptn^−/−^* mice fed with a standard chow diet (STD) or with a high-fat diet (HFD). Data are mean ± SEM of n = 6–12 mice/group. * *p* < 0.05; ** *p* < 0.01; *** *p* < 0.001 for differences in the effect of HFD feeding within *Ptn^+/+^* or *Ptn^−/−^* mice. ^#^ *p* < 0.05, ^##^ *p* < 0.01, ^###^ *p* < 0.001 for differences between *Ptn^+/+^* versus *Ptn^−/−^*.

*Ptn^+/+^* mice had higher liver weight than *Ptn^−/−^* mice and, whereas the administration of a HFD induced a marked increase in liver weight in *Ptn^+/+^* mice, no significant change was observed in the liver weight of *Ptn^−/−^* mice (Figure 2a). Due to the differences in liver weight, we analyzed the hepatic lipid content and the different lipid fractions. *Ptn^−/−^* mice had lower lipid content in liver than *Ptn^+/+^* animals, and this difference was maintained even when mice were fed a HFD (Figure 2b).

This result was confirmed by haematoxylin-eosin staining of liver sections (Figure 2c). Liver sections of HFD-*Ptn^+/+^* mice showed an accumulation of lipid droplets, whereas no accumulation was evident either in the HFD-*Ptn^−/−^* mice, or in *Ptn^+/+^* or *Ptn^−/−^* mice fed a STD.

In the same line of evidence, feeding a HFD increased the content in liver of triacylglycerides, phospholipids, cholesteryl esters and cholesterol in HFD-*Ptn^+/+^* mice when compared to the STD*-Ptn^+/+^* mice. Although HFD also increased these lipid fractions in the *Ptn^−/−^* mice, the values were significantly lower than in wild-type animals fed with a HFD (Figure 2d–g).

### 2.3. High-Fat Diet Feeding along with Ptn Deletion Impairs Circulating Lipid Profile

**Figure 3:**
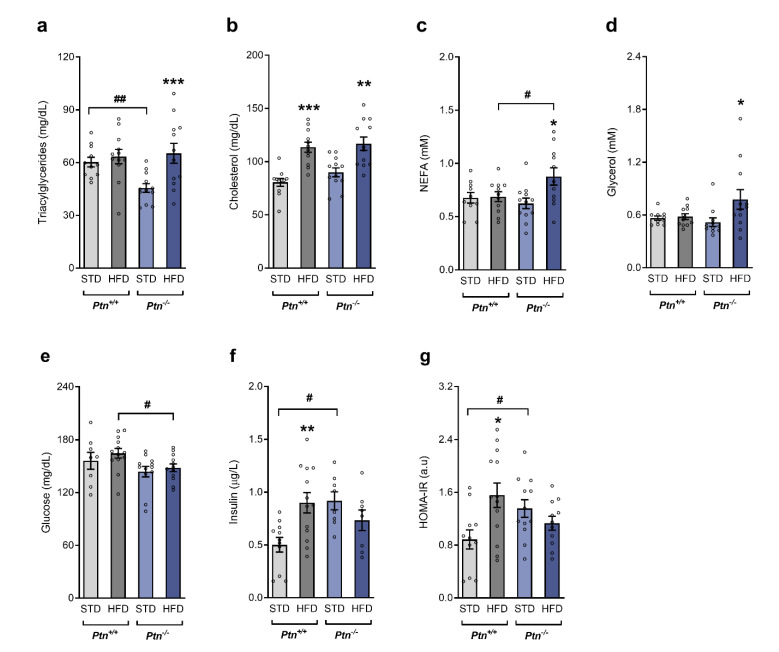
Circulating lipid profile and insulin sensitivity is impaired with high-fat diet and *Ptn* deletion after 6 h fasting. (**a**) Triacylglycerides, (**b**), cholesterol, (**c**) NEFA (**d**) glycerol, (**e**) glucose, (**f**) insulin and (**g**) HOMA-IR index of *Ptn^+/+^* and *Ptn^−/−^* mice fed with a standard chow diet (STD) or with a high-fat diet (HFD). Data are mean ± SEM of n = 8–12 mice/group. *p < 0.05; ** *p* < 0.01; *** *p* < 0.001 for differences in the effect of HFD feeding within *Ptn^+/+^* or *Ptn^−/−^* mice. ^#^ *p* < 0.05; ^##^ *p* < 0.01 for differences between *Ptn^+/+^* and *Ptn^−/−^*.

As shown in Figure 3, after 6 h fasting, triacylglycerides were lower in *Ptn^−/−^* versus *Ptn^+/+^* mice (Figure 3a). Feeding with the HFD in wild-type animals increased circulating cholesterol (Figure 3c), whereas the other fractions remain unchanged. On the contrary, HFD*-Ptn^−/−^* mice exhibited an increase in plasma triacylglycerides, NEFA, cholesterol and glycerol (Figure 3a–d).

No differences in fasting glucose were observed by the effect of the diet in any of the genotypes of mice. However, glycemia was significantly lower in HFD-*Ptn^−/−^* than in HFD-*Ptn^+/+^* mice (Figure 3e). As shown in Figure 3f, fasting insulin was significantly increased in HFD-*Ptn^+/+^* in comparison to STD-*Ptn^+/+^* mice. STD*-Ptn^−/−^* mice had significantly higher fasting insulin levels than STD*-Ptn^+/+^*, and no differences in the insulin levels in *Ptn^−/−^* were observed after 80 days on a HFD (Figure 3f).

As illustrated in Figure 3g, HFD feeding significantly increased the insulin resistance of *Ptn^+/+^* mice (as evidenced by the HOMA-IR). *Ptn* deletion was associated with an insulin resistant state in STD-*Ptn^−/−^* animals to the same extent as that of *Ptn^+/+^* mice after 80 days of a HFD, as evidenced by both the HOMA-IR (Figure 3g) and QUICKI index (an insulin sensitivity index, data not shown), but no significant changes were observed in *Ptn^−/−^* animals by feeding with a HFD.

### 2.4. Effects of High-Fat Diet and Ptn Deletion on Lipid Metabolism in Liver

**Figure 4:**
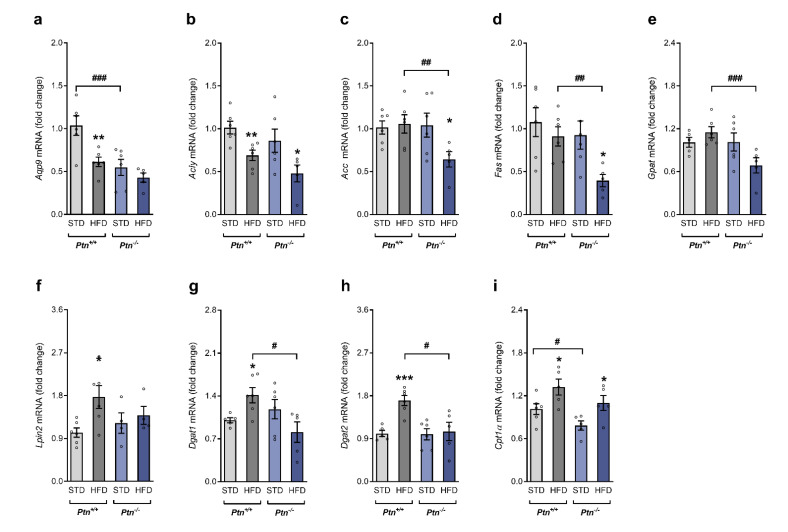
High-fat diet feeding and *Ptn* deletion effects on lipid metabolism in liver. (**a**) *Aqp9* (aquaporin 9) mRNA, (**b**) *Acly* (ATP citrate lyase) mRNA, (**c**) *Acc* (acetyl-CoA carboxylase) mRNA, (**d**) *Fas* (fatty acid synthase) mRNA, (**e**) *Gpat* (glycerol 3-phosphate acyltransferase) mRNA, (**f**) *Lpin2* (lipin 2) mRNA, (**g**) *Dgat1* (diacylglycerol O-acyltransferase 1) mRNA, (**h**) *Dgat2* (diacylglycerol O-acyltransferase 2) mRNA, and (**i**) *Cpt1*α (carnitine palmitoyl transferase 1α) mRNA in the liver of *Ptn^+/+^* and *Ptn^−/−^* mice fed with a standard chow diet (STD) or with a high-fat diet (HFD). Data are mean ± SEM of n = 5–6 mice/group. *p < 0.05, ** *p* < 0.01, *** *p* < 0.001 for differences in the effect of HFD feeding within *Ptn^+/+^* or *Ptn^−/−^* mice. ^#^ *p* < 0.05, ^##^ *p* < 0.01, ^###^ *p* < 0.001 for differences between *Ptn^+/+^* and *Ptn^−/−^*.

We next analyzed the gene expression of key enzymes involved in lipogenesis and triacylglyceride synthesis. As evidenced in Figure 4a,b, the mRNA of *Aqp9* (aquaporin 9) and *Acly* (ATP citrate lyase) were lower in the HFD-*Ptn^+/+^* when compared to the STD-*Ptn^+/+^* mice, whereas no changes were observed in *Acc* (acetyl-CoA carboxylase), *Fas* (fatty acid synthase) and *Gpat* (glycerol 3-phosphate acyltransferase) mRNA (Figure 4c–e). On the other hand, the expression of the genes involved in triacylglyceride synthesis, *Lpin2* (lipin 2), *Dgat1* (diacylglycerol O-acyltransferase 1) and *Dgat2* (diacylglycerol O-acyltransferase 2), were increased in the *Ptn^+/+^* on HFD (Figure 3f–h).

On the contrary, qPCR analysis only revealed a lower expression of *Acly* and *Fas* mRNA in HFD-*Ptn^−/−^* mice when compared to the STD*-Ptn^−/−^* mice. Furthermore, the comparison between genotypes after HFD revealed that the expression of *Fas*, *Gpat*, *Dgat1* and *Dgat2* were significantly lower in the *Ptn^−/−^* when compared to the *Ptn^+/+^* mice. Moreover, the statistical analysis showed interaction in the effects of diet and genotype in both isoforms of *Dgat1* and *Dgat2* (*F* (1, 18) = 8406, and *F* (1, 18) = 6197, respectively). These results may suggest that although HFD feeding is associated in wild-type animals with an increase in hepatic triacylglyceride synthesis, this effect is not observed in *Ptn^−/−^* animals.

We next investigated if the administration of a high-fat diet has any effect in the mRNA of *Cpt1α* (Carnitine palmitoyl transferase 1α), a key enzyme of fatty acid oxidation (Figure 4i). *Ptn* deletion was associated with a decrease in mRNA of *Cpt1α* in the mice fed with STD, whereas *Cpt1α* mRNA was increased by the administration of a HFD in both wild-type and knockout mice.

### 2.5. Fffects of High-Fat Diet and Ptn Deletion on Brown Adipose Tissue UCP-1 Expression

**Figure 5:**
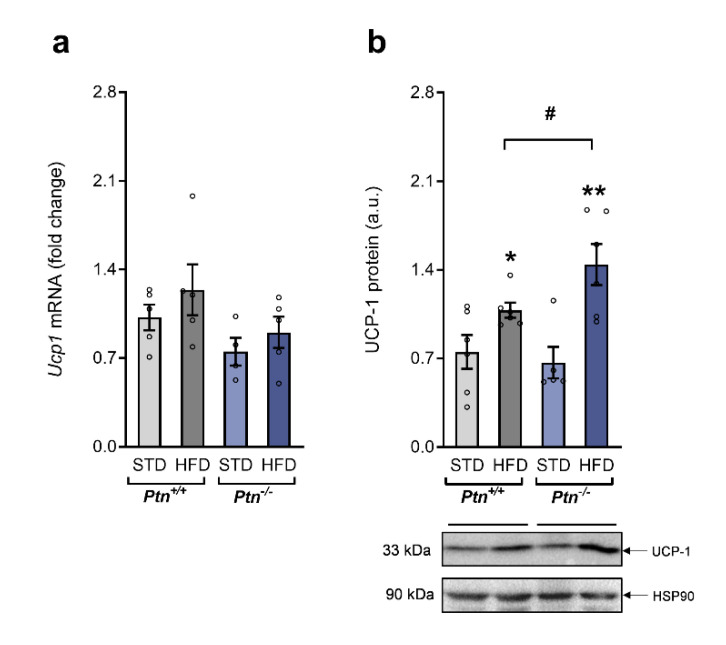
High-fat diet and *Ptn* deletion effects on brown adipose tissue UCP-1*. Ucp1* (uncoupling protein-1) (**a**) mRNA and(**b**) protein in brown adipose tissue of *Ptn^+/+^* and *Ptn^−/−^* mice fed with a standard chow diet (STD) or with a high-fat diet (HFD). Data are mean ± SEM of n = 5–6 mice/group. * *p* < 0.05; ** *p* < 0.01 for differences in the effect of HFD feeding within *Ptn^+/+^* or *Ptn^−/−^* mice. ^#^ *p* < 0.05 for differences between *Ptn^+/+^* and *Ptn^−/−^*.

We next analyzed the effect of *Ptn* deletion and HFD on the expression of UCP-1, the mitochondrial protein responsible for facultative thermogenesis in the brown adipose tissue (Figure 5). *Ucp1* mRNA expression was not modified either by the diet, or the genotype. However, the analysis of the UCP-1 protein levels revealed an increase in UCP-1 protein levels in the mice fed with HFD, that was highest in the BAT of HFD-*Ptn*^−/−^ mice.

### 2.6. Effects of High-Fat Diet and Ptn Deletion on Periovarian AT Browning

**Figure 6:**
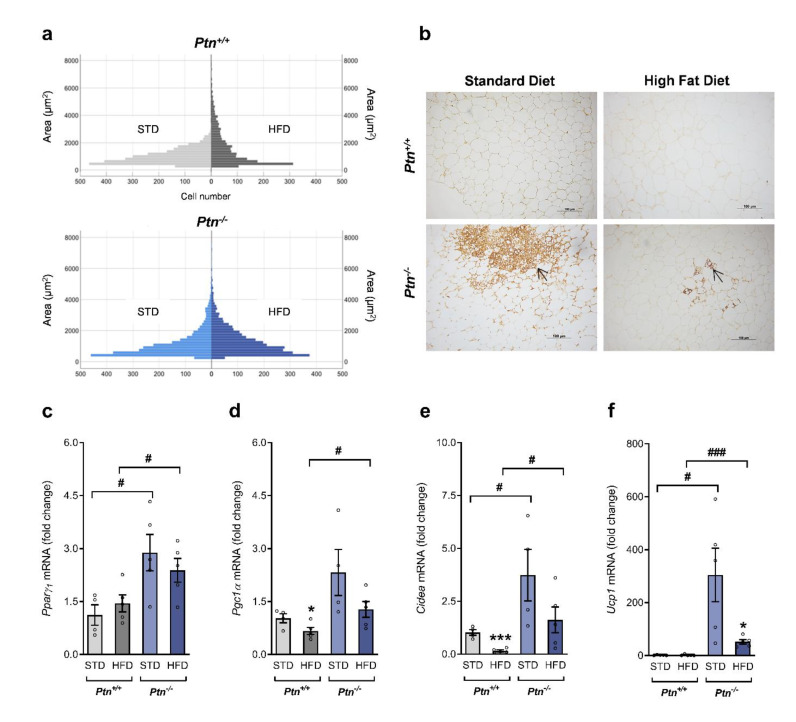
Deletion of pleiotrophin is associated with white adipose tissue browning. (**a**) Adipocyte cell size distribution from the periovarian AT of *Ptn^+/+^* (upper histogram) and *Ptn^−/−^* mice, (bottom histogram). (**b**) Representative immunohistochemistry for UCP-1 in periovarian adipose tissue sections. (**c**) *Pparγ*_1_ (peroxisome proliferator-activated receptor-γ1) mRNA, (**d**) *Pgc1α* (peroxisome proliferator-activated receptor-γ coactivator 1-α) mRNA, (**e**) *Cidea* (cell death-inducing DNA fragmentation factor, alpha subunit-like effector A) mRNA, (**f**) *Ucp1* (Uncoupling protein-1) mRNA in periovarian AT of *Ptn^+/+^* and *Ptn^−/−^* mice fed with a standard chow diet (STD) or with a high-fat diet (HFD). Data are mean ± SEM of n = 4–6 mice/group. * *p* < 0.05, *** *p* < 0.001 for differences in the effect of HFD feeding within *Ptn^+/+^* or *Ptn^−/−^* mice. ^#^ *p* < 0.05; ^###^ *p* < 0.001 for differences between *Ptn^+/+^* and *Ptn^−/−^*. The arrow in panel (**b**) indicates immunoreactivity with UCP-1 antibody.

As we did not observe changes in periovarian adipose tissue weight between both genotypes, the size-frequency distribution of cells was calculated to account for changes in adipocyte size. Comparison by the Mann–Whitney U test (Figure 6a) revealed that a HFD induced in *Ptn*^+/+^ a significant alteration in the cell area distribution (median 856 and 1127 for STD and HFD, respectively, *p* < 0.0001), with a clear increase in the number of big adipocytes (>2000 µm^2^) from 4% in the STD-*Ptn*^+/+^ to 29% in the HFD-*Ptn*^+/+^ mice. Although a HFD also induced a change in adipocyte size distribution in *Ptn*^−/−^ mice, the magnitude of the effect was not so pronounced (median was 873 and 1057 for STD and HFD, respectively, *p* <0.001), and the number of big adipocytes increased from 14% in the mice on the STD to 18% in the animals fed with the HFD (Figure 6a). Furthermore, the size distribution between HFD-*Ptn*^−/−^ mice and HFD-*Ptn*^+/+^ mice was significantly different (*p* < 0.01), indicating that *Ptn* deletion may protect against adipocyte hipertrophy induced by HFD.

Notably, histologic examinations of periovarian adipose tissue sections from STD-*Ptn*^−/−^ mice revealed morphological changes associated with browning, including the appearance of UCP-1 enriched multilocular adipocytes. Similar results were observed in the HFD-*Ptn*^−/−^ mice but with a lower number of clusters of multilocular UCP-1-expressing adipocytes (Figure 6b). To confirm these results, we analyzed by qPCR the mRNA of different markers of adipocytes. As shown in Figure 6c we found that *Ptn* deletion is associated with an increased expression of *Pparg_1_*, independent of the diet. Moreover, *Ptn* deletion was associated with an increase in the expression of *Pgc1α*, *Cidea* and *Ucp1*, specific markers of brown/beige adipocytes, in the periovarian AT of STD-*Ptn*^−/−^ mice (Figure 6d–f). Although the expression of *Pgc1α* and *Cidea* decreased in the *Ptn*^+/+^ when feeding with HFD, the values remained unchanged in the mice lacking *Ptn*. Furthermore, UCP-1 mRNA was more than 100 times higher in *Ptn*^−/−^ mice than in controls, and even if this mRNA decreased after HFD feeding, the values were approximately 60 times higher in HFD-*Ptn^−/−^* when compared to the HFD-*Ptn*^+/+^ mice. Therefore, despite the HFD-induced downregulation of the expression of these brown fat-specific genes, the mRNA levels in HFD*-Ptn*^−/−^ mice were still higher than in the *Ptn*^+/+^ mice.

## 3. Discussion

Obesity is a risk factor that increases the prevalence of hypertension, cardiovascular diseases, NAFLD, insulin resistance and type 2 diabetes [2]. Accordingly, there is an increasing interest in the identification of target molecules involved in the regulation of whole-body energy expenditure, BAT activation and in the modulation of browning of WAT.

Previous studies of our group have shown that pleiotrophin is a key player in the regulation of energy homeostasis and insulin sensitivity [21]. In the present study, we show that *Ptn* deletion increases browning of periovarian white adipose tissue and UCP-1 expression in BAT and ameliorates HFD-induced insulin resistance and liver steatosis.

*Ptn^−/−^* mice have been reported to have smaller body weight and a lower age-related increase in body weight compared with wild-type animals [21]. Here, we show that a HFD increases body weight both in both *Ptn^−/−^* and *Ptn^+/+^* mice, but the increment of body weight was smaller in the *Ptn*-deficient mice [21]. Moreover, although *Ptn* deletion was associated with an increase in the weight of periovarian adipose tissue depots in both genotypes, the HFD-induced increase in adiposity was associated with differences in the adipocyte size distribution and WAT browning.

Adipocyte size and number are indicators of the volume of the lipid content. Obesity is associated with an augmented mass of adipose tissue and an increase in both the number (hyperplasia) and the size of adipocytes (hypertrophy) [25]. Furthermore, changes in the size and distribution of adipocytes have also been linked to diabetes, hepatic steatosis and inflammatory processes [26]. In this study we showed that although HFD is clearly associated in control mice with an altered size distribution of white adipocytes, including a higher proportion of big adipocytes, this hypertrophy of the tissue is partially prevented by deletion of *Ptn*.

Browning of WAT has been shown to significantly increase whole-body energy expenditure and can counteract metabolic diseases, including obesity and type 2 diabetes [27]. Previous studies have identified a population of precursor cells in visceral adipose tissue that can proliferate and differentiate into either UCP-1-expressing adipocytes with adrenergic stimulation, or into white adipocytes with high-fat feeding [28]. In our study, clusters of UCP-1-expressing adipocytes with thermogenic capacity were detected in the periovarian adipose tissue of *Ptn* knock-out mice, but these clusters were almost undetectable in the *Ptn^+/+^* mice. The high expression of a set of brown fat-specific genes (*Ucp1*, *Cidea* and *Pgc1a*) further confirmed the browning of white adipocytes in the periovarian adipose tissue depot of *Ptn* knock-out mice. *Ucp1* and *Cidea* mRNA levels in WAT of HFD-*Ptn^−/−^* mice are downregulated, which may indicate a decline in the number of interspersed brown adipocytes of mice fed a HFD, as has been reported previously [29]. However, despite downregulation with the HFD, the levels of these brown adipocyte markers, particularly UCP-1, are still significantly higher than in STD *Ptn^+/+^* mice.

In vitro experiments with mBAs from our group revealed that BAT differentiation is regulated by changes in *Ptn* expression and suggest an inhibitory role of this cytokine in brown fat differentiation and thermogenesis. Moreover, we have also described that BAT thermogenesis is increased in *Ptn*^−/−^ mice along with an increase in DIO2 activity and expression, higher concentrations of T3 in BAT and subsequently lower levels of plasma-free T4 [21]. In fact, expression and activation of UCP-1 is stimulated by T3, and DIO2 is required for the conversion of T4 to T3 [30].

Here, an increased brown adipose tissue UCP-1 protein content was observed in both genotypes after feeding a HFD, but the increment in UCP-1 protein was higher in the HFD-*Ptn^−/−^* mice. The increase in UCP-1 levels was associated with a decrease in the levels of plasma-free T4 (data not shown), indicative of the increased conversion to T3 in BAT, as we have observed before in this mouse model [21]. The increase in UCP-1-mediated thermogenesis in BAT has been commonly reported as a compensatory mechanism to increase energy expenditure and prevent diet-induced obesity, although the increases in the expression have been reported to be very variable in magnitude [29].

The increase in UCP-1 protein expression in BAT and the browning of WAT in the *Ptn^−/−^* mice, even when fed with a HFD, may be associated with an elevated thermogenesis and may also account for the reduced ectopic lipid deposition. In fact, HFD-induced accumulation of lipids in the muscle (data not shown) is reduced in the *Ptn^−/−^* mice when compared to the control animals. Moreover, our data show that *Ptn^−/−^* mice are resistant to HFD-induced steatosis, as evidenced by the reduced hepatic lipid accumulation as a consequence of the decrease in the content of triacylglycerides, cholesteryl esters and cholesterol in the liver. This connection between periovarian adipose tissue and liver is not surprising since this WAT depot, similar to other visceral fat, is characterized by its direct communication to the liver via the portal vein [31]. Additionally, the lower lipid accumulation in the liver data can be explained by the reduced expression of the enzymes involved in hepatic lipid synthesis. Although glycerol and fatty acid esterification is favored in *Ptn^+/+^* mice on a HFD, as evidenced by the increase in *Lpin2*, *Dgat1* and *Dgat2* expression, this effect is not observed in *Ptn^−/−^* mice. In fact, *Ptn* deletion is associated in the liver with decreased mRNA of the enzymes involved in both the triacylglyceride synthesis (*Gpat*, *Lpin2*, *Dgat1* and *Dgat2)* and fatty acid synthesis (*Acc* and *Fas*).

Additionally, as the plasma levels of triacylglycerides are increased in HFD-*Ptn^−/−^* mice when compared to STD-*Ptn^−/−^*, we cannot discard the possibility that an increased hepatic secretion of triacylglycerides as very-low-density lipoproteins (VLDL) may contribute to reduce the hepatic lipid content in the HFD-*Ptn^−/−^* mice and provide substrates for oxidation in both white and brown adipose tissue. Furthermore, plasma levels of glycerol and NEFA are also increased in HFD-*Ptn^−/−^* mice, indicating an increased lipoprotein catabolism or increased lipolysis of triacylglycerides in adipose tissue.

In agreement with our previous study, at 6 months of age STD-*Ptn^−/−^* mice exhibited an altered plasma biochemical profile, with lower plasma glucose levels, higher insulin concentration and higher HOMA-IR than control mice, which suggests an amelioration of insulin sensitivity in these animals [21]. Moreover, although the administration of a HFD induced hyperinsulinemia and insulin resistance in the HFD-*Ptn^+/+^* mice, neither insulinemia nor the insulin resistance index increased in HFD-*Ptn^−/−^* mice, suggesting a protective role of pleiotrophin deletion in the HFD-induced insulin resistance.

Altogether, these data suggest that *Ptn* deletion may protect against the development of HFD-induced steatosis and whole-body insulin resistance, by promoting browning of WAT and an enhanced thermogenesis in white and brown fat.

## 4. Materials and Methods

### 4.1. Animals

PTN genetically deficient mice (*Ptn^−/−^*) were generated as previously described [32]. C57BL/6J female wild-type (*Ptn^+/+^*) and *Ptn^−/−^* mice were divided randomly and housed at 22–24 °C with 12h light/dark cycles, and free access to water and chow (STD, 18 kcal% fat, 58 kcal% carbohydrates and 24% kcal protein; 3.1 kcal·g^−1^) or a high-fat diet (HFD, D12451, 45 kcal% fat, 35 kcal% carbohydrates and 20% kcal protein; 4.73 kcal·g^−1^) as corresponding. Diet administration was maintained for 80 days, and animals were fed ad libitum. After 6 h of fasting, 6-month-old mice from each experimental group (n = 11–13) were killed by decapitation under CO_2_ exposure. Plasma and tissues were collected and stored at −80 °C. All the animals were maintained under European Union Laboratory Animal Care Rules (2010/63/EU directive) and protocols were approved by the Animal Research Committee of CEU San Pablo University and by Comunidad de Madrid (PROEX 137/18).

### 4.2. Plasma Analysis

Glucose ((GOD-PAP); Roche Diagnostics, Barcelona, Spain), triacylglycerides ((LPL- GPO); Roche Diagnostics), cholesterol ((CHOD-POD); Spinreact, Girona, Spain), glycerol (GPO-Trinder, Sigma Diagnostic, Madrid, Spain) and NEFA ((ACS-ACOD); Wako Chemicals, Neuss, Germany) levels were determined by enzymatic colorimetric tests. Plasma insulin measurement (Mercodia, Uppsala, Sweden) was determined using immunoassay kits. The HOMA-IR insulin resistance index and QUICKI insulin sensitivity index were calculated as previously described [33].

### 4.3. Histology

Fixed liver sections (4 μm) were dehydrated and then embedded in paraffin. The sections were deparaffinized, rehydrated and stained with haematoxylin and eosin (H&E) to examine the morphology of the tissue. Sections were analysed by optical microscopy (Leica Biosystems, Barcelona, Spain).

### 4.4. Hepatic Lipid Analysis

Total lipids were extracted and purified by the Folch method [34]. Different lipid fractions were separated by thin-layer chromatography in Silicagel for quantification.

### 4.5. Immunohistochemistry Analyses

Fixed adipose tissue sections (4 μm) were dehydrated and then embedded in paraffin. The sections were deparaffinized, rehydrated and incubated with primary antibody UCP-1 (Abcam, Cambridge, UK). Sections were incubated with a biotinylated anti-IgG (Vector Laboratories, Burlingame, CA, USA) and incubated with the avidin–biotin–peroxidase complex (Vector Laboratories). 3,3′-diaminobenzidine (DAB) substrate (Merck, Darmstadt, Germany) was used as the chromogen. Some samples were incubated without primary antibody as negative controls. The stained adipose tissue sections were imaged with a Zeiss Standard 25 light microscope.

To measure the area of adipocytes, four slides per animal were quantified (n = 4 animals/group), and non-consecutive slides were taken. Image J 1.45 software (National Institutes of Health, Bethesda, MD, USA) and Adiposoft program (http://fiji.sc/Adiposoft) were used.

### 4.6. Quantitative Real-Time PCR

Total RNA was isolated using the Total RNA Isolation Kit (Nzytech, Lisbon, Portugal) and the first-strand cDNA was synthesized using the first-strand cDNA Synthesis Kit (Nzytech). Quantitative real-time PCR analysis was performed using the SYBR green method (Quantimix Easy kit, Biotools, Madrid, Spain) in a CFX96 Real Time System (Bio-Rad, Hercules, CA, USA). The relative expression of each gene was normalized using *Hprt* and *Rpl13* as reference genes. The primer sequences are shown in Table 1.

### 4.7. Protein Extraction and Immunoblotting

Brown adipose tissue was homogenized in a pH 7.4 buffer containing 30 mM HEPES, 5 mM EDTA, 1% Triton X-100, 0.5% sodium deoxycholate, 8 mM Na3VO4, 1 mM NaF, and 2 mM of the protease inhibitor mixture Pefabloc (Roche Diagnostics, Barcelona, Spain). Protein concentration was measured using the Pierce BCA protein method (Thermo-Scientific, Waltham, MA, USA).

An equal amount of protein was subjected to SDS-PAGE electrophoresis, transferred to PVDF membranes (Amersham-GE Healthcare, Amersham, UK), and incubated with anti-UCP-1 primary antibody (Merck, Kenilworth, NJ, USA). HSP90 (Merck) was used as the loading control. After the incubation with the corresponding horseradish peroxidase-conjugated secondary antibody (Sigma-Aldrich, St. Louis, MO, USA), proteins were visualized by the enhanced chemiluminescence (ECL) system (GE Healthcare, Chalfont Saint Giles, UK) using the ChemiDoc XRs Imaging system (Bio-Rad, Hercules, CA, USA) and quantified by densitometry.

### 4.8. Statistical Analysis

Results are expressed as mean ± SEM. Normality was assessed by the Shapiro–Wilk test, and a Grubbs’ test was run to detect outliers. Comparisons between two groups were made by Student’s *t*-test for equal or unequal variances. When data were not normally distributed a log transformation was performed before analysis. Statistical analysis was conducted using GraphPad Prism v8 (USA). Size-frequency distribution of adipose cells was compared by the non-parametric Mann–Whitney U test using IBM-SPSS v27. The differences in the effect of HFD feeding within *Ptn^+/+^* or *Ptn^−/−^* mice are indicated with asterisks. The differences between *Ptn^+/+^* and *Ptn^−/−^* are indicated with hashtags.

## Figures and Tables

**Table 1:** Primer sets used for qPCR analysis.

Gene	Primer Forward	Primer Reverse
*Acc*	5′-GTCCCCAGGGATGAACCAATA-3′	5′-GCCATGCTCAACCAAAGTAGC-3′
*Acly*	5′-AAGCCTTTGACAGCGGCATCATTC-3′	5′-TTGAGGATCTGCACTCGCATGTCT-3′
*Aqp9*	5′-CTATGACGGACTCATGGCCTTT-3′	5′-ATGAACGCCGTTCCATTTTCT-3′
*Cidea*	5′-GCCTGCAGGAACTTATCAGC-3′	5′-AGAACTCCTCTGTGTCCACCA-3′
*Cpt1α*	5′-ACCCTGAGGCATCTATTGACAG-3′	5′-ATGACATACTCCCACAGATGGC-3′
*Dgat 1*	5′-GCCCCATGCGTGATTATTGC-3′	5′-CACTGGAGTGATAGACTCAACCA-3′
*Dgat 2*	5′-AACCGAGACACCATAGACTACTT-3′	5′-CTTCAGGGTGACTGCGTTCTT-3′
*Fas*	5′-AGAGATCCCGAGACGCTTCT-3′	5′-GCCTGGTAGGCATTCTGTAGT-3′
*Gpat*	5′-ACGCACACAAGGCACAGAG-3′	5′-TGCTGCTCAGTACATTCTCAGTA-3′
*Hprt*	5′-TGCTCGAGATGTCATGAAGG-3′	5′-TATGTCCCCCGTTGACTGAT-3′
*Lpin2*	5′-AGTTGACCCCATCACCGTAG-3′	5′-CCCAAAGCATCAGACTTGGT-3′
*Pgc1α*	5′-CCCTTCTTTGCCATTGAATC-3′	5′-AATGTTAGGAAAGTTTAGCATCTGG-3′
*Rpl13*	5′-GGTGCCCTACAGTTAGATACCAC-3′	5′-TTTGTTTCGCCTCCTTGGGTC-3′
*Ucp1*	5′-GGATTGGCCTCTACGACTCA-3′	5′-TAAGCCGGCTGAGATCTTGT-3′

## Data Availability

Data associated with this study is available upon reasonable request.
